# Decoding hybrid origins and genetic architecture of leaf traits variation in *camellia* via high-density 21K SNP array for genomic prediction

**DOI:** 10.1093/hr/uhaf221

**Published:** 2025-08-22

**Authors:** Jiayu Li, Yixuan Luo, Rui Zhang, Xinchun Li, Hongwei Pan, Hengfu Yin

**Affiliations:** State Key Laboratory of Tree Genetics and Breeding, Zhejiang Key Laboratory of Forest Genetics and Breeding, Research Institute of Subtropical Forestry, Chinese Academy of Forestry, Hancgzhou, Zhejiang Province 311400, China; College of Landscape and Tourism, Hebei Agricultural University, Baoding, Hebei 071000, China; State Key Laboratory of Tree Genetics and Breeding, Zhejiang Key Laboratory of Forest Genetics and Breeding, Research Institute of Subtropical Forestry, Chinese Academy of Forestry, Hancgzhou, Zhejiang Province 311400, China; State Key Laboratory of Tree Genetics and Breeding, Zhejiang Key Laboratory of Forest Genetics and Breeding, Research Institute of Subtropical Forestry, Chinese Academy of Forestry, Hancgzhou, Zhejiang Province 311400, China; College of Landscape and Tourism, Hebei Agricultural University, Baoding, Hebei 071000, China; Tianjin GenoAgri Company Co., Ltd, Tianjin 300385, China; State Key Laboratory of Tree Genetics and Breeding, Zhejiang Key Laboratory of Forest Genetics and Breeding, Research Institute of Subtropical Forestry, Chinese Academy of Forestry, Hancgzhou, Zhejiang Province 311400, China; State Key Laboratory of Tree Genetics and Breeding, Zhejiang Key Laboratory of Forest Genetics and Breeding, Research Institute of Subtropical Forestry, Chinese Academy of Forestry, Hancgzhou, Zhejiang Province 311400, China

## Abstract

The domestication of ornamental plants is primarily driven by aesthetic values and usually involves frequent hybridization events. *Camellia* spp., a globally famous woody flower, exemplifies the complex origins and extensive phenotypic variation. Here, based on the whole genome resequencing 220 germplasms, we developed Camellia21K, a high-density SNP array enabling cost-effective genome-wide genotyping. We demonstrated that Camellia21K accurately resolves 69 cultivars with complex hybridization histories. For molecular identification of closely related varieties, we developed a set of fingerprinting SNPs to support variety discrimination. To dissect the genomic basis of ornamental traits, we performed a genome-wide association study (GWAS) analysis of five leaf shape traits using the Camellia21K array and screened 31 SNP loci significantly associated with the traits. Further, by analyzing the genotypes of the SNP loci and the haplotypes of the surrounding segments, we identified potential genes regulating leaf tip length, thus demonstrating the versatility of the array. To enhance breeding efficiency, we evaluated and optimized four genomic selection (GS) models for leaf trait prediction. We found that the number of SNPs and model selection significantly affected prediction performance, with optimal predictive accuracy (PC) from 0.362 to 0.542, which was positively correlated with heritability. Finally, we integrated fixed-effects SNPs from GWAS and found significant enhancement of PC (24.7%–64.7%), indicating that the combination of GWAS and GS is indispensable for precision breeding applications. We demonstrated that Camellia21K is effective in discriminating the origin of varieties, in genetic analysis of traits and in genomic prediction, and thus informative for crop breeding.

## Introduction

Unlike many crops, the breeding of ornamental plants is primarily driven by the selection of aesthetic values and is usually not constrained by genetic bottlenecks associated with economic traits such as yield [1]. To create innovative ornamental cultivars with distinctive characteristics, breeders commonly rely on identifying and incorporating key genetic mutations, as well as performing interspecific and intraspecific hybridizations among diverse genotypes [2]. These strategies are highly effective in generating desirable phenotypic variations, but often lead to cultivars with complex genetic backgrounds, complicating efforts to uncover the genetic mechanisms underlying the formation of key ornamental traits [3]. For example, in roses, after a long period of selection and extensive hybridization, modern hybrid roses (*Rosa hybrida*) have displayed remarkable diversity in ornamental traits, such as flower forms, colors, and fragrances, with about 20 original rose species contributing to their genetic background [4]. However, in the process of analyzing these trait variations, the origins of genetic variation and their regulatory mechanisms appear particularly complex due to the influence of events such as hybridization, backcrossing, and polyploidization.

Genomics research in ornamental plants has expanded significantly in recent years, driven by advancements in sequencing technologies and computational tools. Nowadays, reference genomes are available for almost all representative ornamental plants or close relatives, and the growing number of transcriptome and population resequencing studies provide a rich resource for elucidating the breeding origins of varieties and the formation of ornamental traits [5, 6]. For example, *Prunus mume* was probably the first woody flower to complete a reference genome [7]; subsequent studies have used population resequencing and association analyses to identify key regulatory genes associated with traits such as flower color, floral shape, and plant architecture [6, 8]. However, the analysis of the complex genetic backgrounds of many ornamental varieties and the interpretation of trait variations are often limited by existing population analysis methods [9]. Therefore, on the basis of reference genomes, how to develop cost-effective technologies suitable for population differentiation, trait analysis, and prediction remains a key challenge in current breeding research.

Single-nucleotide polymorphism (SNP) arrays are a well-established, high-throughput genotyping tool that identifies the genotype of a target locus with a specifically designed probe [10]. Recent studies have demonstrated that the development of high-density SNP arrays provides an unprecedented role in accelerating genetic research and molecular breeding of crops [11, 12]. Targeted sequencing genotyping (GBTS) is a high-throughput genotyping technique that combines second-generation sequencing technology with the use of primers or probes designed to capture specific DNA fragments [13]. Compared with traditional solid-phase microarrays, GBTS technology is simpler and more economical. Moreover, GBTS utilizes deep sequencing technology with fixed target sites, which provides accurate and reproducible results and facilitates data integration and sharing [14, 15]. GBTS technology has been widely used in the field of crop breeding, and a variety of liquid-phase microarrays with different densities have been developed based on the GenoBaits platform [16–18]. For example, liquid-phase SNP arrays, such as ZJU CottonSNP40K [19], Cassava35K [20], and Melon2K [21] have been demonstrated to provide high-resolution genomic insights, resolve complex traits and hybridization histories, and facilitate trait association studies and genomic selection (GS). In forest trees, especially those with extraordinarily large and complex genomes such as *Pinus massoniana* [22], *Pinus elliottii* [23], and *Picea abies* [24], high-density SNP arrays have a pivotal role in advancing the GWAS and GS of growth, disease resistance, wood quality, and other traits [22–24].

*Camellias* (*Camellia* spp.) is a collective term for the varieties and germplasm of the genus *Camellia* that have been bred primarily for ornamental purposes and are now found all over the world. Ornamental *Camellia* spp. varieties come from a wide range of sources, and there is a wealth of variability in ornamental traits, such as floral shape, color, bloom duration, and fragrance [25–27]. Among them, some *Camellia* varieties with breakthrough ornamental traits are often the result of interspecific hybridization. For example, the *Camellia sasanqua* series of varieties, yellow *Camellias* (derived from *C. nitidissima*, etc.), ever-blooming *Camellias* (derived from *C. azalea*) [28] and fragrant *Camellias* (derived from *C. lutchuensis*, etc.) [29], are all fantastic ornamental varieties with interspecific hybridization sources. These abundant genetic variations not only provide valuable genetic resources for enhancing the ornamental value of camellias and developing new varieties but also introduce complexity and challenges in trait analysis and targeted genetic improvement.

In recent years, genomic research on the genus *Camellia* has made rapid progress. Reference genome sequences of economically important crops such as tea (*C. sinensis*) and oil-Camellia (*C. oleifera*) have been published [30, 31]. Tea plants have generated a wealth of genomic resources, such as TeaPGDB, reflecting the economic and agricultural importance of tea as a major non-alcoholic beverage and the role of its unique secondary metabolites in determining quality traits [32, 33]. Recently, a versatile SNP array named TEA5K has been developed, enabling efficient and high-throughput genotyping to support marker-assisted selection and genetic diversity studies in tea [34]. Currently, the genomic information of some ornamental camellias has also been elucidated. For instance, the genomes of representative species such as *C. japonica* ‘Naidong’ [35], *C. chekiangoleosa* [36], and *C. limonia* (golden camellias) [37] have been successfully decoded. Although a 200 000 SNP array has been developed in *C. sinensis* for high-density genetic map construction and origin analysis [38], high-throughput SNP genotyping platforms for ornamental camellias are lacking. In order to dissect the genetic background of important ornamental traits, it is of great significance to develop efficient SNP analysis techniques to explore and analyze the genomic resources of ornamental camellias. Here, we developed Camellia21K through integrating genomic variations from diverse sources of *Camellia* varieties and showed that the SNP array is proficient in analyzing fine-scale population structures and tracing the origins of modern varieties, interspecific hybrids, and geographically divergent cultivars. We further performed and optimized the GWAS and GS applications on five leaf morphology traits, which provide valuable insights for genetic breeding of perennial ornaments.

## Results

### Characteristics of the Camellia21K SNP array and validation of efficiency

**Figure 1:**
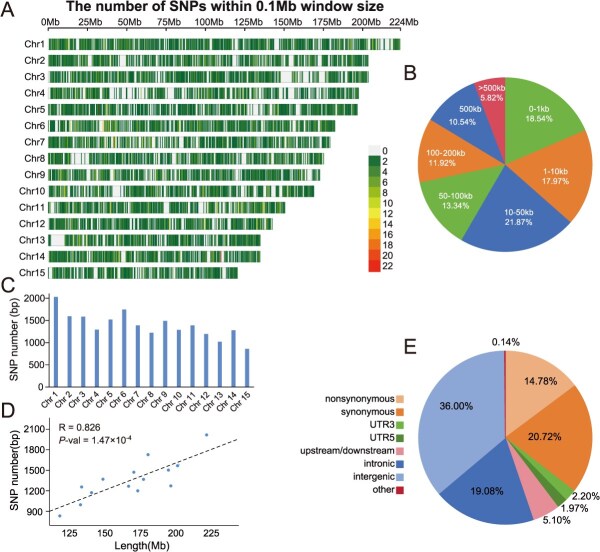
Characteristics of the Camellia21K SNP array. (A) Density of SNP markers within 0.1-Mb windows across the 15 chromosomes. The colors indicate the range of SNP density. (B) A piechart of the distribution of distances between adjacent SNPs. (C) The number of SNPs on each chromosome. (D) The relationship between number of SNPs on the chromosome and chromosome length. (E) Distribution of number of SNPs based on the genome annotation information.

To develop a SNP array for ornamental *Camellia* breeding, we combined the genome resequencing data from 220 varieties with SNPs identified from a genetic linkage map. We applied stringent filtering and probe design criteria to select high-quality, unique SNPs suitable for array construction (see Materials and Methods for details). We examined the distribution of SNPs in the pseudo-chromosomes of the *C. japonica* genome and found that they were uniformly distributed (Fig. 1A). The average distance between adjacent SNPs was 123.40 kb, with only 5.82% (1216) pairs of SNPs having a distance greater than 500 kb (Fig. 1B). We evaluated the number of SNPs on each chromosome and showed that the number of SNPs was correlated with chromosome length (Fig. 1C and D), indicating an even distribution of SNPs on each chromosome. We revealed that 19.08% SNPs were located in the intergenic regions, and there were 14.78% and 20.72% SNPs nonsynonymous and synonymous in the coding regions, respectively (Fig. 1E).

To access the efficacy of the SNP array, we performed Camellia21K-targeted sequencing by using 15 representative samples with diverse origins (Table S1). We observed that the Camellia21K array demonstrated high capture efficiency for target fragments across a wide range of germplasm accessions, with an average mapping rate of 98.26%, an average sequencing depth of 83×, and an average coverage of 96.37% (Table S1). The targeted sequencing of Camellia21K array was designed to hybridize to their corresponding genomic regions which enabled the selective capture of both the target SNP sites and their surrounding flanking sequences After applying stringent quality filters (missing rate < 0.2, minor allele frequency (MAF) ≥ 0.05, and retaining only biallelic loci), a total of 477 173 high-quality SNPs were identified. We further checked the recovery accuracy of targeted SNPs (20 900 SNPs) and found that that an average of 19 650 sites were successfully detected per sample, with a detection rate of 94.02% (Table S1). To assess the reproducibility of the SNP array, we compared the genotyping results of 14 samples that were analyzed using both genome resequencing and the Camellia21K SNP array. We found a high level of consistency between the two methods, with an average concordance rate of 92.6% (Table S1). Based on these results, we conclude that the Camellia21K array is an efficient and reliable tool for accurate genotyping of target SNPs.

### Population structure and phylogenetic relationship analysis based on the Camellia21K SNP array to distinguish the origin of *Camellia* cultivars

**Figure 2:**
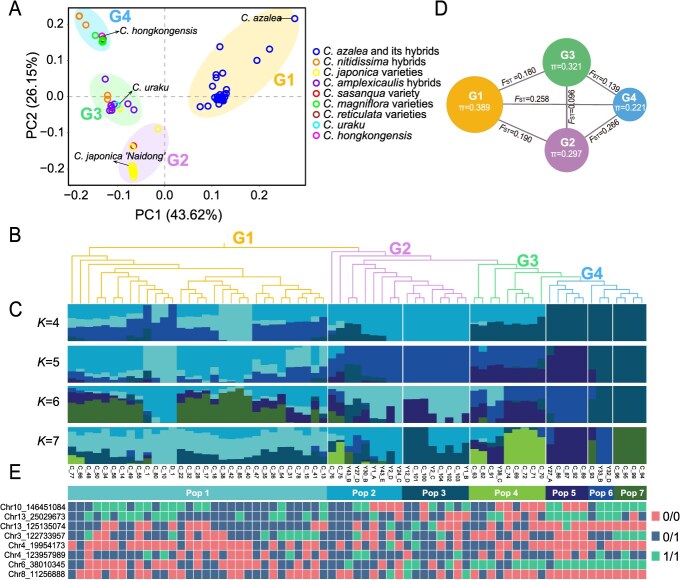
Population structure, genomic diversity and fingerprints of 69 *Camellia* accessions. (A) The first two components of Principal Component Analysis (PCA) illustrate the four groups of 69 camellia accessions, with different color circles representing different varieties and different background colors representing different groups. (B) A neighbor-joining phylogenetic tree is constructed based on filtered SNPs. The four major groups are indicated by different colors that correspond to those in the PCA analysis. (C) Population structure analysis results for *K* = 4–7. The y-axis quantifies cluster membership, and the x-axis represents different accessions. (D) Nucleotide diversity (π) and population divergence (*F*_ST_) across the four groups. The size of circles is on scale with π of each group. (E) Fingerprints of the 69 *Camellia* accessions. Each row represents the typing result of the same SNP in different accessions, and each column represents an accession. Pink, homozygote genotypes identical to the reference genome (0/0); green, homozygote genotypes different from the reference genome (1/1); blue, heterozygous genotypes (0/1). The location information of the samples is consistent in B, C, and E, and the sample accessions are listed in C.

The ornamental *Camellias* are diverse and often have hybrids from closely related species. To clarify the origin of *Camellia* cultivars, we used the genome-wide Camellia21K array to perform genotyping and population analyses. A total of 69 *Camellia* ssp. accessions were selected for array analysis based on their representative origins, 15 of which were used in the aforementioned array evaluation (Table S2). Among the 69 samples, 30 *C. azalea* hybrids, 6 *C. nitidissima* hybrids, and 8 *C. amplexicaulis* hybrids were known for their hybrid origin; while for the modern *Camellia* varieties, their origins are less clear (Table S2). By using the Camellia21K array for sequencing analysis of 54 samples, we observed that all samples exhibited high capture efficiency, with an average mapping rate of 98.24%, an average sequencing depth of 77×, and an average coverage of 96.39% (Table S3). For the targeted SNP loci, an average of 19 380 SNPs were successfully detected per sample, corresponding to a marker detection rate of 92.73% (Table S3). Following stringent filtering of all captured SNP loci across the samples (missing rate = 0, MAF ≥ 0.15), a total of 60 370 high-quality SNPs were obtained and used for subsequent genetic analyses. The PCA analysis revealed four major population clusters (G1 to G4) (Fig. 2A), and otably, all *C. azalea* hybrids clustered together with the original *C. azalea* species (Fig. 2A). Additionally, hybrids from *C. amplexicaulis* also appeared to cluster together (Fig. 2A), demonstrating high resolution in analyzing the genetic origins of the varieties.

We further integrated the phylogeny and genomic admixture analyses to dissect the sample relationship (Fig. 2B and C). We showed that seven subgroups (*K* = 7) combined with phylogenetic relationships showed the best classification of samples, in consideration of recorded information of cultivars (Fig. 2B and C). For example, G2 and G3 were dominated by *C. japonica* cultivars, including wild species of *C. japonica* ‘Naidong’ and *C. uraku* (Fig. 2B and C). In G4, the *C. amplexicaulis* hybrids were clustered with two *C. nitidissima* hybrids, reflecting a close relationship among parental species (Fig. 2B and C). To assess the genetic diversity of the cultivar groups, we analyzed the *F*_ST_ and π values for the four groups. Our findings revealed that G1 exhibited the highest genetic diversity (π = 0.389), which is consistent with its composition of all *C. azalea* hybrids. Furthermore, we observed substantial genetic differentiation between G4 and both G1 and G2, with *F*_ST_ values of 0.258 and 0.266, respectively (Fig. 2D). These findings suggest that G4 represents a more distantly related evolutionary lineage.

To investigate the complexity of the genetic background, we evaluated samples showing a single dominant admixture (*K* = 7) in each subgroup; these samples were difficult to separate on the basis of phylogetic categorization. To achieve an accurate determination, we performed additional selection of SNPs for molecular determination of varieties by KSAP (kompetitive allele-specific PCR) analysis. The common SNPs (9194) from Camellia21K of samples were extracted and evaluated for primer design analysis, which yielded 97 suitable SNPs located in exonic regions (Table S4). Following the principle of ‘distinguishing the most varieties with the fewest SNPs’ [39], we identified a set of 8 SNPs capable of distinguishing all varieties, which led to the construction of a core SNP set for fingerprinting (Fig. 2E).

**Figure 3:**
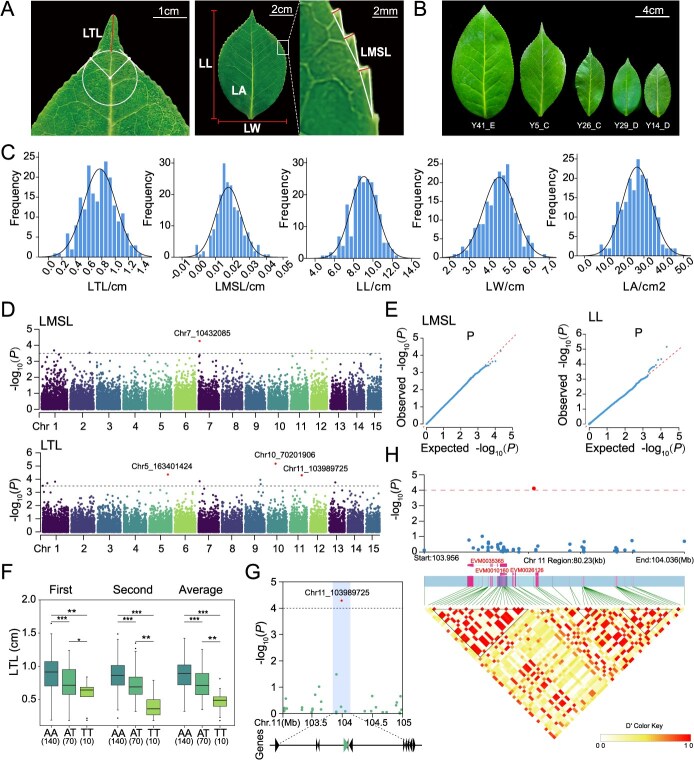
Genome-wide association study (GWAS) using the Camellia21K SNP array for characterizing leaf shape variations. (A) Measurement of five leaf shape traits. The red lines indicate LTL, LL, LW and LMSL. LTL: leaf-tip length; LMSL: leaf-margin serration length; LL: leaf length; LW: leaf width; LA: leaf area. For LTL, the inner tangent circle of the tangent line to the turning point on either side of the leaf tip was determined and the distance from the leaf tip to the circle was measured along the direction of the leaf veins. For LMSL, the upper one-third region of the leaf blade was selected for measuring (magnified area indicated by the white box), the average length of three consecutive serrations was measured. (B) Leaf morphology of six *Camellia* accessions with high degree of variability (C) Histograms of the measurements of five leaf traits, with fitted normal distribution curves for each trait by black lines. (D) Manhattan plot of GWAS results for LTL and LMSL. The black horizontal dashed line represents the threshold level of significance –log_10_(*P*) = 3.5, and the significant SNPs are marked by red. (E) The quantile–quantile (Q–Q) plot of all expected and observed *P*-values for LTL (left) and LMSL (right). (F) Leaf-tip length for three different haplotypes based on the genotype at Chr11_103989725. For the box plot, the horizontal line in the box indicates the median value, the box height indicates the 25th to 75th percentiles of the total data, the whiskers indicate the interquartile range. ^*^P < 0.05; ^**^P < 0.01; ^***^P < 0.001. (G) Local Manhattan plot of 2 mb regions of Chr.11 containing the 400 kb genomic region (blue shade) surrounding Chr11_103989725, and the gene models of 12 candidates in the shaded regions are presented (the triangles on the bottom). (H) LD heatmap of the region around the SNP locus Chr11_103989725. The SNPs and gene annotation are presented on top, and colored lines indicate the relative positions of genes and SNPs. On the bottom, the degree of association between SNPs was measured by the D value and presented by colored map.

### GWAS analysis of leaf shape traits based on the Camellia21K SNP array identifies key candidate genes and genetic loci

To evaluate the utility of SNP arrays in uncovering genetic variation in ornamental traits, we measured five leaf traits of varieties in the resequencing population for two times, including LL, LW, LA, LTL and LMSL (Fig. 3A and B). We found that the measured leaf phenotypes showed significant variation within the population, and the distribution of all trait variations followed a typical normal distribution (Fig. 3C; Table S5). Through the single-marker mixed linear model (EMMAX), we performed GWAS analysis for these leaf traits. A total of 31 significant SNPs were obtained at a threshold of −log_10_(*P*) = 3.5 (Fig. 3D and E; Fig. S1; Table S6). Under the more stringent threshold line −log_10_(*P*) = 4, three SNPs significantly associated with leaf tip length and one SNP significantly associated with leaf margin serration length were identified (Fig. 3D). To evaluate the effectiveness of the Camellia21K array, we compared the GWAS results obtained using SNP data from whole-genome resequencing. By analyzing the *P* values of 31 significant SNPs, we found that the results from the Camellia21K array were significantly correlated with those derived from resequencing data using both EMMAX and GEMMA analysis methods (Fig. S3). The correlation coefficient (R) reached 0.685 when analyzed under same EMMAX parameters, demonstrating the reliability and utility of the Camellia21K array for GWAS in *Camellia* species.

**Figure 4:**
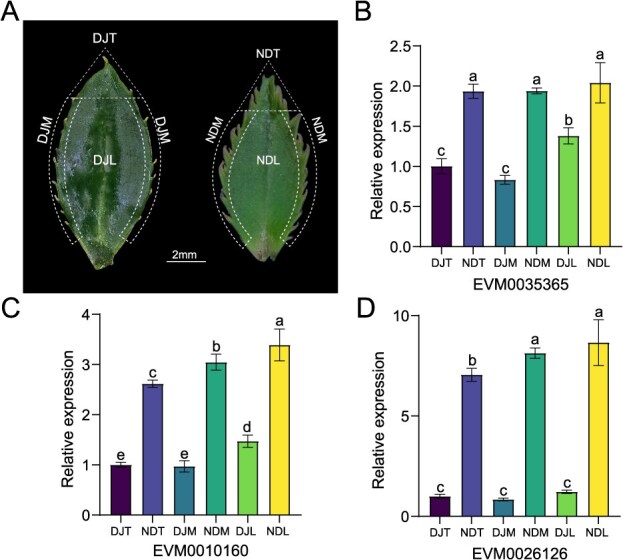
Differential expression analysis of candidate genes in different parts of the leaf. (A) Leaf morphology of *C. azalea* (left) and *C. japonica* ‘Naidong’ (right). (B) Differential expression analysis of EVM0035365, EVM0010160, and EVM0026126 in different parts of the leaf of *C. azalea* and *C. japonica* ‘Naidong’. The significance of differences was tested using ANOVA, and different letters represent significant differences (*P* < 0.05). Data are mean ± S.D (*n* = 3 for three biological repeats). DJT: The leaf tip of *C. azalea*; NDT: The leaf tip of *C. japonica* ‘Naidong’; DJM: The leaf margin of *C. azalea*; NDM: The leaf margin of *C. japonica* ‘Naidong’; DJL: The leaf lamina of *C. azalea*; NDL: The leaf lamina of *C. japonica* ‘Naidong’.

We further investigated the association between the leaf trait variations and the haploid types of the significant SNPs (Fig. 3F; Fig. S2). We found that, Chr11_103989725 was significantly associated with LTL (*P* = 5.18 × 10^−5^), and the three haplotypes (AA, AT and TT) at this position showed significant differences in both measurements as well as in the average values, with the AA haplotype corresponding to the longest leaf tip, followed by AT, and lastly TT (Fig. 3F). We then evaluated the genes in the 200 kb region upstream and downstream of the locus for functional annotation (Table S7). A total of 12 genes were annotated in the 200 kb region upstream and downstream of the SNP locus (Fig. 3G). The region was then haplotyped using 488 747 SNPs, resulting in 7 LD blocks (Fig. 3H). The largest block covers a 9.824 kb region (containing a total of 25 SNP) and there are three candidate genes (including EVM0035365, EVM0010160 and EVM0026126) closely located around the region. (Fig. 3H).

To evaluated the three candidate genes, we carried out gene expression analysis by using incipient leaves of *C. azalea* and *C. japonica* ‘Naidong’, two *Camellia* plants showing distinct differences in leaf morphology (Fig. 4A). By comparing expression patterns across three distinct leaf regions—the leaf tip, leaf margin, and central leaf region—we found that the expression levels of all three candidate genes were significantly different between *C. japonica* and *C. azalea* (Fig. 4B–D). Among these, EVM0035365 is located 8291 bp from the significant SNP and encodes a protein phosphatase belonging to the PP2C gene family [40]. Studies have shown that several members of the PP2C family are involved in leaf shape development through direct or indirect regulation of meristem activity and stem cell proliferation in plant meristematic tissues, suggesting a broader role in leaf development [41, 42]. Taken together, these results indicate that the Camellia21K SNP array is a valuable tool for GWAS analysis, enabling the identification of key SNP loci and candidate genes associated with trait variation.

### Optimization of GS model analysis using leaf-related traits offers possibilities for precision breeding

**Figure 5:**
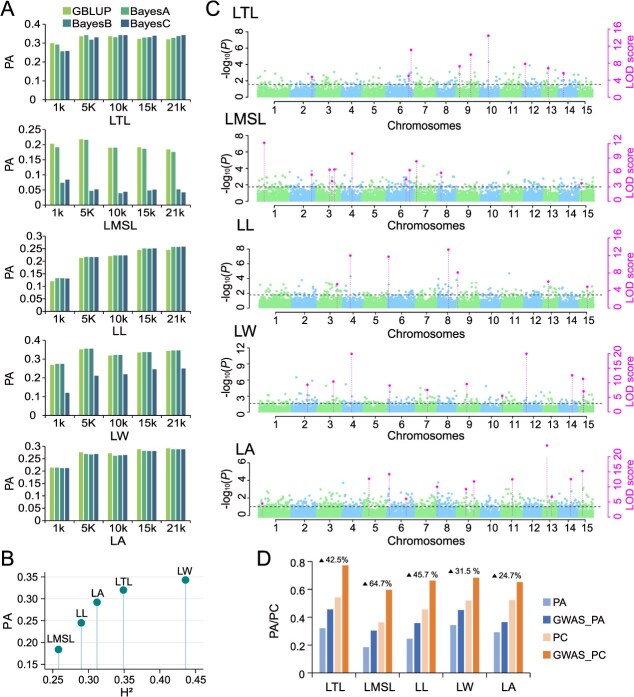
Optimization and evaluation of genomic prediction models based on the Camellia21K SNP array. (A) Predictive ability of four prediction models (GBLUP, BayesA, BayesB and BayesC) for five leaf traits based on randomly selected SNP datasets of 1 K, 5 K, 10 K, 15 K, and the full 21 k SNPs. (B) The relationship between the heritability of traits and their GS predictive ability. (C)Manhattan plots of main effect QTN for five traits based on the IIIVmrMLM model. The left Y-axis reports the −log10 *P*-value for the main effect QTN, which was obtained in the first step after a single-marker genome-wide scan of all markers; the right Y-axis reports the LOD score, which was obtained in the second step after a likelihood-ratio test of the significant suggested QTNs, with a threshold of LOD = 3.0 (dashed line). (D) Comparison of the impact of adding significant SNPs from GWAS results as Fixed Effects on the Predictive Ability (PA) and Prediction Accuracy (PC) for five traits using the GBLUP model. The percentages of enhancement of model performance for each trait are presented on the top.

To test the utility of the SNP array for trait prediction, we implemented GS models using five-fold cross-validation for predicting the five leaf traits. We systematically evaluated the effects of four statistical models (GBLUP, BayesA, BayesB, and BayesC) and five SNP subsets of varying sizes (1 K, 5 K, 10 K, 15 K, and 21 K) on prediction accuracy (PA). The results showed that the PA of all models using 1 K SNPs was relatively low (Fig. 5A). For models using 5 K to 21 K SNPs, the changes of PA values were minor, with higher SNP densities generally leading to higher PA values overall (Fig. 5A). The choice of GS model had a significant impact on different phenotypes. For instance, BayesC performed poorly in predicting LW and LMSL, whereas GBLUP consistently demonstrated high PA across all five traits (Fig. 5A).

Heritability (H^2^) was shown to play a crucial role in determining the PA of GS [43]. Given the short runtime and high efficiency of GBLUP model, we examined the correlation between PA of GBLUP and H^2^. A significant positive correlation (*R* = 0.894; *P* = 0.041) was observed between the heritability of traits and their GS predictive ability (Fig. 5B), which suggested that the Camellia21K array effectively captured the genetic variations contributing to leaf-related traits. To improve the accuracy of GS predictions, we incorporated GWAS results as fixed effects into the GS model. Previous studies have shown that GS models integrating *de novo* GWAS findings can significantly enhance trait prediction accuracy and accelerate genetic gain in plant breeding programs [44]. In this study, we employed the multi-locus GWAS method IIIVmrMLM, which simultaneously models multiple SNPs and estimates their joint effects. The IIIVmrMLM approach first conducts a genome-wide scan using a permissive significance threshold to identify potentially associated SNPs. These candidate markers are then included in a multi-locus framework, where significant quantitative trait nucleotides (QTNs) are detected through empirical Bayesian estimation combined with likelihood ratio tests [45]. Based on this multi-locus GWAS analysis, a total of 50 QTNs significantly associated with five leaf morphology traits were identified (Fig. 5C). These QTNs, along with the significant SNPs previously identified through single-marker GWAS (Table S8), were integrated as fixed effects into the GBLUP model. We showed that the PA and PC of the models for all traits were significantly improved (24.7%–64.7%). Among them, the model optimization of LA traits was the most significant, with an improvement of 64.7% (PA: 0.184–0.303; PC: 0.362–0.597) (Fig. 5D). Thus, our results illustrate that effective GS prediction requires the integration of key loci information from GWAS results.

**Figure 6:**
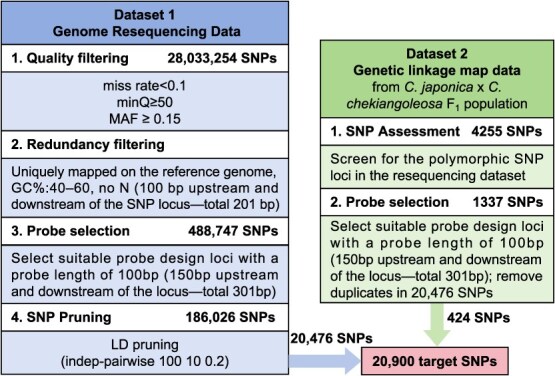
The flow diagram for SNP selection and design of Camellia21K SNP array. The blue box illustrates the details of filtering process for 28 033 254 SNPs from the resequencing dataset; and the green box represents the filtering process for 4255 SNPs from the genetic linkage map. The resequencing data went through four filtering processes, resulting in 20 476 high-quality SNPs. From the genetic linkage data, SNPs were cross-referenced to identify polymorphic variants that were also present in the resequencing dataset, resulting in a subset of 1337 SNPs. Following probe selection and the removal of redundant SNPs, 424 high-quality SNPs were incorporated into the final SNP array. The red box represents the final set of target SNPs for probe design.

## Discussion

### The Camellia21K SNP array is a significant advancement for molecular breeding of ornamental camellias

In crop plants, high-throughput SNP marker genotyping technology, which not only contains tens of thousands of markers distributed throughout the genome, but also allows the analysis of a large number of samples, is of great significance for its application in breeding-related aspects [46]. Modern ornamental *Camellia* varieties may contain genetic background sources from dozens of *Camellia* spp [47]. In order to reveal the genetic basis of ornamental traits and provide a basis for molecular breeding, it is important to establish effective SNP genotyping techniques. In recent years, advances in ornamental *Camellia* genomics research have provided a rich resource for better understanding the variation in various ornamental traits [35]. However, the development and application of large amounts of genetic and genomic data, which are crucial for improving breeding efficiency, have not been sufficient. By integrating genome-wide SNPs from resequencing data and genetic linkage mapping, the Camellia21K array provides a high-throughput, high-resolution, and cost-effective genotyping method. We designed a rigorous screening process including quality filtering, redundancy removal, probe optimization, and SNP pruning to ensure the high efficiency of the markers (Fig. 6); through validation testing of the samples, we found that the detection rate of target SNPs averaged more than 94% for both intraspecific and interspecific samples (Fig. 1). These results are comparable to the current performance of SNP arrays in multiple crops (e.g. Melon2k, Cassava35K) [20, 21]. The use of SNP loci to identify species and cultivars of ornamental plants is important for plant breeding applications [48, 49]. In order to make Camellia21K compatible with fingerprinting analysis, we have optimized genotyping protocols (e.g. KASP marker) to easily and accurately identify varieties in the face of complex hybrids and highly similar varieties. For the 69 germplasm materials in this study, it was possible to distinguish all cultivars using the 8-SNP core set (Fig. 2E), which highlights the utility of the array for intellectual property protection and hybrid identification in breeding programs.

For crops with multiple interspecific genetic backgrounds, a single representative genome may be biased due to loss of species-specific genetic information; and identifying unique variations from genomes of different parents or pan-genomic information from multiple individuals can improve the precision of the genomic analyses [50]. For example, the CottonSNP63K array containing 45 104 intraspecific SNPs and 17 954 interspecific SNPs was validated with 1156 samples, resulting in two high-density genetic maps corresponding to *Gossypium raimondii* and *G. hirsutum*, respectively, which facilitated trait analysis and genomic selection [51]. In our work, the Camellia21K chip, currently using *C. japonica* as a single reference genome, may not be representative of some hybrids with typical parental groups. For example, we found that the genetic backgrounds of most *C. azalea* hybrids were complex and divergent from the other groupings (Fig. 2B–E), suggesting that selection of SNP loci that are more parent-specific may improve the accuracy of resolution. Therefore, one of the goals of future research is to construct and expand SNP arrays based on the genomes of wild relatives and their pan-genomes. In addition, the breeding of *Camellia* varieties often faces a large number of polyploid germplasm [52], and it is also important to develop a ‘haploid-aware’ SNP genotyping system.

### Camellia21K facilitates genomic dissection of leaf shape variation

Leaf shape is not only an important ornamental feature but also plays a crucial role in plant survival, adaptation, and classification [53, 54]. The variation in leaf shape is largely determined by genetic factors [54, 55]. Numerous regulatory genes have been identified as being involved in the establishment of leaf polarity, the formation of leaf edge serrations, and the generation of specialized leaf structures [56, 57]. Across diverse plant species, subtle changes in the functions of these conserved regulatory genes—such as differences in their expression patterns or protein functions—have been shown to result in the emergence of diverse leaf morphologies [58]. For example, based on research in *Arabidopsis*, the formation of leaf margin serration patterns has been demonstrated to be determined by PIN-FORMED (PIN)-mediated auxin export and the localized expression of *CUP-SHAPED COTYLEDON2* (*CUC2*) [59]; and this process is co-regulated by various phytohormones and transcription factors, such as TCP and *KNOX* (Class I KNOTTED1-like homeobox) family members [60]. Here, we measured five leaf shape traits in ornamental *Camellia* varieties. Despite the diversity of leaf morphology among varieties, it still exhibited high morphological stability within genotypes (Fig. 3A–C). The heritability of the five leaf traits we determined ranged from 0.25 to 0.45 (Fig. 5B), making them suitable for population genetic analysis. Leaf morphology is strongly influenced by environmental factors. For example, in poplar, leaf morphology can be highly diversified and variable during different developmental stages as well as in different growth environments [61, 62]. To accurately capture the genetic factors underlying leaf variation, this study employed an image analysis-based method to efficiently quantify five key traits for two independent times. We found that the two datasets were highly correlated, and by integrating the data, environmental interference could be effectively reduced (Fig. 3F).

Our GWAS analysis based on the Camellia21K platform identified four high-priority significantly associated SNPs: one linked to LTL and three to LMSL (Fig. 3D). Haplotype analysis at these loci revealed significant associations between phenotypic effects and specific alleles (Fig. 3F). We identified three candidate genes within the associated genomic regions (Fig. 4). Through gene expression analysis across different leaf morphotypes in *Camellia*, we observed significant variation in the expression patterns of these candidate genes (Fig. 4). Notably, through functional evaluation, we identified the PP2C family phosphatase gene EVM0035365 as a key candidate gene involved in leaf development (Fig. 3G and H). Previous research has demonstrated that PP2C genes are involved in regulating meristematic activity, a conserved function in leaf development [63]. Taken these, the Camellia21K array serves as a powerful tool for elucidating the genetic architecture of ornamental traits in *Camellia*. In future work, our primary objective is to enhance the accuracy of genetic analysis for complex ornamental traits. To achieve this, we aim to integrate high-throughput tools such as LeafAnalyser [64] to expand population size and increase population diversity, thereby improving phenotype–genotype association analyses.

### GS prediction enhanced by GWAS-integrated models highlights breeding potential

The development of GS has significantly promoted the progress of forest tree breeding and effectively solved the key problems such as long cycle time, low efficiency and limited application of new technologies [65]. By utilizing high-throughput genotyping technologies, such as gene chips, low-cost, flexible, and accurate genotyping has been achieved [66], laying the foundation for the wide application of GS. Substantial progress has been made in microarray-based GS analysis in a variety of crop and forest species [67, 68].

It has been shown that the PA of GS is affected by a variety of factors, such as the size of the training population, the prediction model, marker density, and trait heritability [69]. Given that GBLUP performs well and efficiently with Bayesian models (BayesA, BayesB, and BayesC) in GS [70, 71], we compared the PA of four GS models and five SNP marker sizes for leaf traits using the Camellia21K SNP array (Fig. 5A). The results showed that the choice of model and the size of marker sets were crucial for the PA of leaf traits (Fig. 5A). We found that, the GBLUP model is more consistent and well-performed among the leaf traits (Fig. 5A), indicating its stability and applicability in leaf shape traits. It has been found that leaf phenotypes are usually controlled by minor-effect polygenes [72], which explains the good results of our GBLUP model. It also suggests that the SNP array of Camellia21K is an effective representation of genome-wide SNP-based cumulative effects for predicting complex leaf traits [70]. For traits controlled by a small number of dominant genes (e.g. specific disease resistance), Bayesian models (e.g. BayesC) are more appropriate because they allow for heterogeneity in the variance of marker effects [73].

By comparing the number of SNPs, we found that the PA tended to stabilize when the number of markers reached 21K (Fig. 5A), indicating that the number of SNPs had reached the saturation point for the trait. This indicates that the density of this SNP array is sufficient to comprehensively capture the genetic variation of the target trait, and thus the current SNP density achieves a good balance between performance and economy.

Further, the strong positive correlation (*R* = 0.894) between trait heritability and PA confirmed the ability of the array to capture additive genetic variation (Fig. 5B) [43]. Notably, integrating GWAS-derived QTNs as fixed effects in the GS model significantly improved PA and PC (Fig. 5C). Especially for the LMSL trait with low heritability, the PA improvement reached a surprising 64.7% (Fig. 5D), thus demonstrating that the integration of GWAS for GS prediction in response to complex and environmentally sensitive traits is a crucial step. Based on this approach, the Camellia21K SNP array accelerates the breeding cycle by prioritizing markers with known phenotypic effects and efficiently identify superior genotypes, making it a powerful tool for research and breeding of camellias.

## Conclusions

In this study, we constructed the Camellia21K SNP array and elucidated its versatility, including high-throughput genotyping, identification of hybrids by KASP, GWAS-driven gene discovery, and GS-optimized trait prediction. By linking genotypes to phenotypes, the SNP genotyping platform helps address long-standing challenges in camellias breeding, such as the genetic complexity associated with interspecific hybridization and the need for rapid breeding of new varieties. Our work also provides insights into molecular breeding for multiple complex agronomic traits in crops.

## Materials and methods

### Plant materials

The samples of *Camellia* resources in this study were preserved in the *Camellia* Germplasm Resource Conservation Center of the Reseach Institute of Subtropical Forestry (RISF), Chinese Academy of Forestry (119.96°E, 30.06°N), of which 15 *Camellia* samples were used to test the efficiency of the SNP array; and 220 germplasms were used for leaf trait measurement and genome resequencing to carry out GWAS and GS analyses. In order to conduct multi-source genetic structure and phylogenetic analyses, 54 new *Camellia* varieties were originally collected from Guangdong Province, Guangxi Autonomous Region, Hunan Province and Yunnan Province, and also kept at RISF. Genomic DNA of all samples was prepared from fresh leaves using cetyltrimethylammonium bromide (CTAB) by the magnetic-bead mediated method, and high-quality DNA (1.8 < OD260/280 < 2.0, OD260/230 > 2.0) was used for target sequencing.

### The pipeline of SNP selection for the development of Camellia21K SNP array

To develop a SNP array for ornamental *Camellia* breeding applications, we implemented a selection pipeline based on two SNP datasets of *C. japonica*, including SNPs from resequencing dataset of 220 camellias varieties and SNP makers from a genetic linkage map [35] (Fig. 6). The genome resequencing SNPs were processed through four main steps as follows: (1) Quality filtering: The 28 033 254 raw SNPs obtained from genome resequencing were filtered using VCFtools (v.1.40) [74] by applying the following criteria: missing rate < 0.2, minimum quality score (minQ) ≥ 50, MAF ≥ 0.15, and exclusion of SNPs located on unplaced contigs. (2) Redundancy filtering: The SNP region (100 bp upstream and downstream), characterized by a GC content ranging from 0.4 to 0.6 and no ‘N’ bases, was mapped to the *C. japonica* reference genome [35] to check the sequence redundancy. Sequences are considered duplicates if they have a similarity of 60% or more and align to more than 60% of the genomic sequence; and a total of 488 747 SNPs were retained as unique. (3) Probe selection: To design probe for SNP, we selected 150-bp sequences upstream and downstream of the SNP locus and use a window size of 100 bp and a step size of 1 bp to evaluate all potential probes. The probe sequences with a GC content between 40% and 60%, no duplicate sequences (as described in step 2), and no ‘N’ bases were retained as qualified. In total, 186 026 suitable SNPs were obtained for probe design. (4) SNP Pruning: Lastly, we performed SNP pruning using PLINK (v.1.90) [75] (−indep-pairwise 100 10 0.2), resulting in 20 476 SNPs for array design. The SNPs used for the genetic mapping were processed and integrated into the design of the SNP microarray through the following two steps: (i) SNP Assessment: A total of 4255 SNPs were initially obtained from the genetic linkage map data of the *C. japonica* × *C. chekiangoleosa* F_1_ hybrid population. To identify polymorphic SNPs that were also present in the resequencing dataset, cross-referencing was performed, resulting in a subset of 1337 SNPs selected for further analysis. (ii) Probe Selection: Screen for SNPs suitable for probe design using the same method as before. A total of 424 high-quality SNPs met these criteria and were combined with 20 476 previously selected SNPs, resulting in a final set of 20 900 SNPs used in the design of the Camellia21K array (Fig. 6; Table S9).

### SNP array construction and SNP calling process

Liquid-phase probe synthesis and sample capture sequencing were performed at Tianjin GenoAgri Company (Tianjin, China). The overall process was as described before [23], and the main steps are as follows: (i) Genomic DNA was fragmented (200–300 bp), added to sequencing adapters, and pre-PCR amplified to construct sequencing libraries. (ii) The specific probes were designed based on an optimized thermodynamic stability algorithm model. The probe is later used to hybridize to the target area. (iii) Streptavidin-coated magnetic beads were used to capture the hybridized probe-target complexes. (iv) The enriched target fragments were eluted and subjected to post-PCR amplification after capture. (v) Target region sequencing was performed on the DNBSEQ-T7 high-throughput sequencing platform. The raw sequences are openly available in National Genomics Data Center at https://www.cncb.ac.cn/ (BioProject ID: PRJCA039435).

SNP calling and filtering went through the following process: (i) Raw reads are processed through adapter cutting, high N content filtering, and low-quality base filtering to generate clean reads for subsequent analyses. (ii) Clean reads for each sample were then mapped to the reference genome using BWA (v.0.7.17) [76]. By comparing and locating the position of clean reads on the reference genome [35], the sequencing depth, genome coverage and other information of each sample were calculated. (iii) Individual VCF (variant call format) calls were performed by the Genome Analysis Toolkit (GATK v.4.1.2.0) [77] based on the HaplotypeCaller method, followed by population SNP calls by using GenomicsDBImport and GenotypeGVCFs by merging all the VCFs. To filter SNPs, the Variant Filtration (hard filter command) process was used to exclude potential false-positive variant calls with parameters as following: QD < 2.0, MQ < 40.0, FS > 60.0, SOR > 3.0, MQRankSum < −12.5, QUAL <30.0 and ReadPosRankSum < −8.0.

### Phylogenetic and population structure analyses

The phylogenetic analysis was performed using VCF2Dis (v.1.53) [78] to calculate genetic distances. TreeBest (v.1.9.2) [79] software was used to construct individual-based neighbor-joining (NJ) trees based on genetic distances with 1000 bootstrap replications, and then the phylogeny was visualized by iTOL [80]. The Principal Component Analysis (PCA) analysis was performed using the PLINK (v.1.90) [75] software and plotted using the R package ggplot2 (v.3.5.1) [81]. Population structure was analyzed using ADMIXTURE software (v.1.3.0) [82] and visualized using the R package pophelper (v.2.3.1) [83]. Nucleotide diversity (π) and fixation index (*F*_ST_) were calculated by VCFtools (v.0.1.16) [74]. The core set of SNPs that could distinguish all varieties for fingerprinting analysis was calculated by SNPT software (http://www.shigatox.net/stec/cgi-bin/snpt).

### Leaf phenotypic analysis and statistical analysis

For sampling and leaf trait analysis, three mature leaves were collected from the middle portion of annual branches of each tree, and high-resolution photographs were taken to capture leaf morphology. This approach has been demonstrated as an effective sampling strategy in previous studies of species such as poplar [84], cotton [85], and alfalfa [86], to capture leaf morphological variation for genetic association studies. Two independent sampling experiments were conducted in May and November 2024. For each individual, three leaves were selected to calculate average leaf characteristics per genotype, ensuring representative phenotypic measurements while minimizing within-plant variation. The leaf tip length (LTL), leaf margin serration length (LMSL), leaf length (LL), leaf width (LW), and leaf area (LA) were measured by using ImageJ [87]. LTL is measured as follows (Fig. 3A): first find the leaf tip turning point (the part where the leaf tip meets the leaf margin), then make the tangent circle of the tangent line of the two points, follow the direction of the leaf veins from the very tip of the leaf to the distance of the circle that is the length of the leaf tip, and the mean value of the three leaves was recorded. For LMSL (Fig. 3A), at one-third of the blade, three serrations were selected for length measurements, then the shortest distance from the tip to the bottom edge of the serration was measured as the length of the serration, and finally the mean of a total of nine values from the three leaves was recorded.

### GWAS analysis

GWAS analyses were performed using the mixed linear model (MLM) by EMMAX [88]. To avoid false positives, the Kinship Matrix, a pairwise genetic similarity matrix based on Simple Matching Coefficients calculations, was used as the Variance–Covariance Matrix for the random effects; and the first 3 PCs of PCA was also constructed as fixed effects in the model for the effects of population structure. The Manhattan plot of all SNPs was evaluated to find the most significant SNPs for following analyses. Quantile–Quantile (Q–Q) plots were also used for validation in order to estimate the difference between observed and predicted values for each quantitative trait. To determine the significance threshold for GWAS, we used Plink [75] to estimate the number of independent markers across the genome. This was done by applying SNP pruning with the parameters –indep-pairwise 100 10 0.1 to obtain the effective number of independent SNPs (*N*). The genome-wide significance threshold was then calculated as −log_10_(1/*N*) = 3.63. To balance the risk of false positives and false negatives, we set the final significance threshold at −log_10_(*P*) = 3.5 for identifying significantly associated SNPs. For the GWAS analysis of the resequencing results, EMMAX and GEMMA [89] software were used as previously described. SNPs were filtered for quality and LD before identifying significant associations. The LDBlockShow software (v.1.40) was used for the linkage disequilibrium (LD) calculation between SNPs and visualization [90].

### Quantitative real-time PCR (qRT-PCR) for gene expression analysis

Leaf tip, leaf margin, and leaf lamina tissues were collected from two representative *Camellia* plants—*C. azalea* and *C. japonica* ‘Naidong’—which has distinct leaf morphologies. Total RNA was extracted from the collected plant tissues using the HiPure HP Plant RNA Mini Kit (Magen, Guangzhou, China), followed by reverse transcription using TakaRa RR036A PrimeScript™ RT Master Mix. Gene-specific primers were designed using Primer Express® software (v.3.0.1) (Table S10). The qRT-PCR was performed on a QuantStudio™ 7 Real-Time PCR System with TakaRa RR820A TB Green® Premix Ex Taq™ II. The relative expression levels of candidate genes were calculated using the 2^−ΔΔCt^ method, with CjGAPDH employed as the internal reference gene.

### GS analysis

Genomic prediction for five leaf-related traits was conducted based on 220 *Camellia* samples. GBLUP is implemented through the ‘mmer’ function of the sommer (v.4.3.7) package in R [91]. The GBLUP statistical model is:


$$ \mathrm{y}=\mathrm{X}\mathrm{\beta} +\mathrm{Z}\mathrm{\mu} +\mathrm{\varepsilon} $$

where y is a vector of phenotypic values, β is a vector of fixed effects, μ is a vector of random effects, X and Z are incidence matrices for fixed and random effects respectively, ϵ is the residual. BayesA, BayesB, and BayesC are implemented using the ‘BGLR’ function in the R package BGLR (v.1.1.2) [92]. Bayes class generalized models for:


$$ \mathrm{y}=\mathrm{X}\mathrm{\beta} +\mathrm{W}{\mathrm{a}}_{\mathrm{m}}+\mathrm{\varepsilon} $$

where y is a vector of phenotypic values, β is a vector of fixed effects, X is the association matrix for β, W is the matrix of SNP marker genotypic scores, a_m_ is the SNP marker random effect, and ϵ is the error effect [93]. A 5-fold cross-validation method was used, where the reference population was randomly divided into 5 subsets, 4 of which were taken as the training population and the remaining 1 as the validation population, and this process was repeated 100 times [94]. The predictive ability (PA) was assessed by calculating Pearson correlation coefficients between the phenotypic values and the predicted genomic breeding values (GEBVs) of the validation population. The GBLUP model was used to estimate the variance components for each trait and to calculate the broad-sense heritability (H^2^):


$$ {\mathrm{H}}^2=\frac{\sigma_{\mathrm{g}}^2}{\left({\mathrm{\sigma}}_{\mathrm{g}}^2+{\mathrm{\sigma}}_{\mathrm{e}}^2\right)} $$

where H^2^ is the broad-sense heritability, ${\mathrm{\sigma}}_{\mathrm{g}}^2$ is the genetic variance, ${\mathrm{\sigma}}_{\mathrm{e}}^2$ is the error variance. The prediction accuracy (PC) was assessed using the following formula: PA/√H^2^.

The population was analyzed using the multi-locus analysis model 3VmrMLM based on the IIIVmrMLM (3 Variance-component multi-locus random-SNP-effect Mixed Linear Model) software (v.1.0) package to find Quantitative Trait Nucleotides (QTNs) that were significantly associated with the target traits [45]. This model uses a compressed variance component mixed model approach to assess additive and dominant effects, as well as their interactions with the environment and epistatic effects, ensuring comprehensive control of all potential polygenic backgrounds. The model threshold is set to LOD = 3.

## Supplementary Material

Web_Material_uhaf221

## Data Availability

All raw sequencing data are publicly accessible through the National Genomics Data Center at https://www.cncb.ac.cn/ under BioProject ID: PRJCA039435. All associated data are provided in the supplementary materials of the manuscript, and all codes used in the analysis are also available upon request.
