# Supplementary figures and images for: Decoding hybrid origins and genetic architecture of leaf traits variation in camellia via high-density 21K SNP array for genomic prediction
Source: Hortic Res. 2025 Aug 22;12(11):uhaf221. doi: 10.1093/hr/uhaf221 (PMC12581787; doi:10.1093/hr/uhaf221)

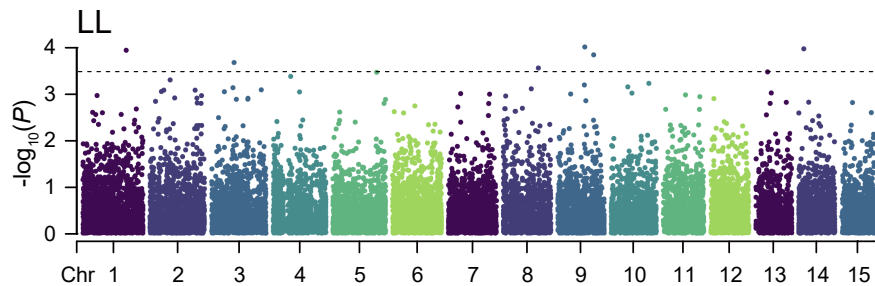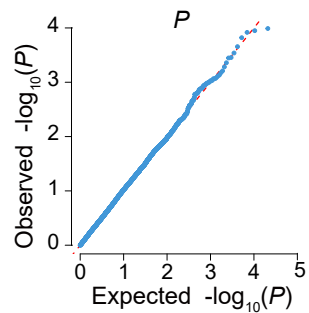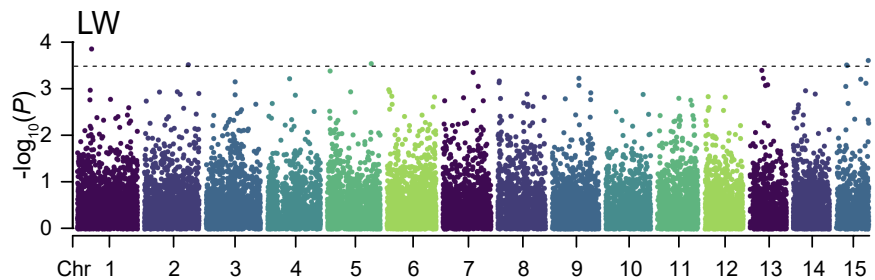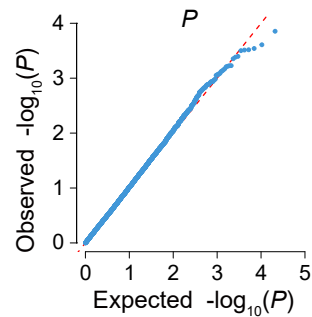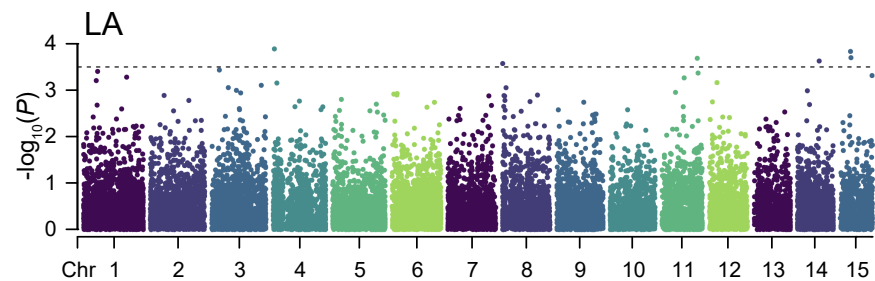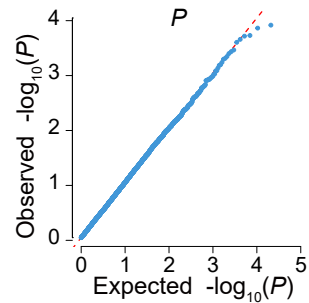

Supplement: Web_Material_uhaf221 [file web_material_uhaf221.zip › fig S1.pdf]

Chr 5\_163401424

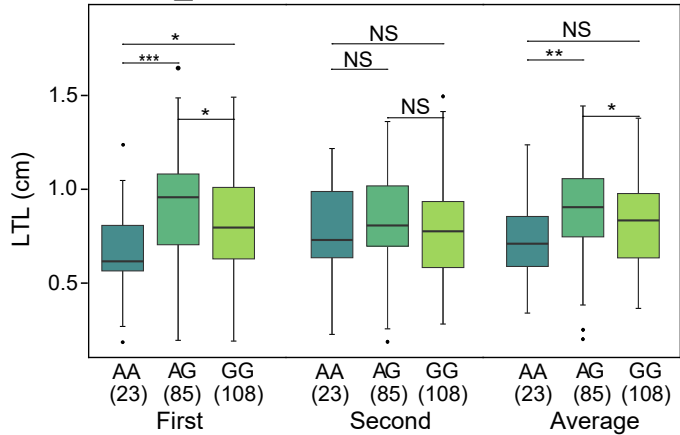

Chr 10\_70201906

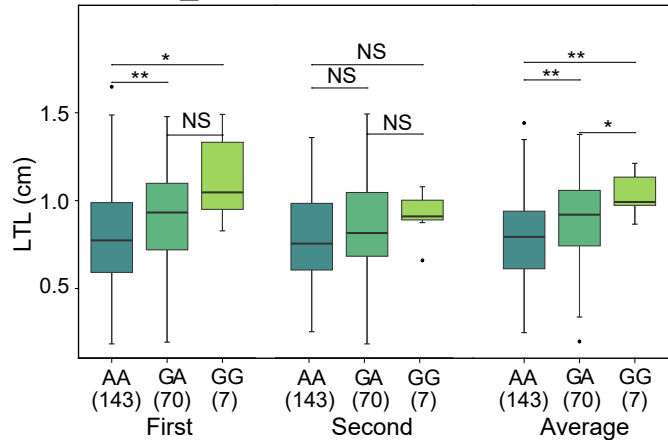

Supplement: Web_Material_uhaf221 [file web_material_uhaf221.zip › fig S2.pdf]

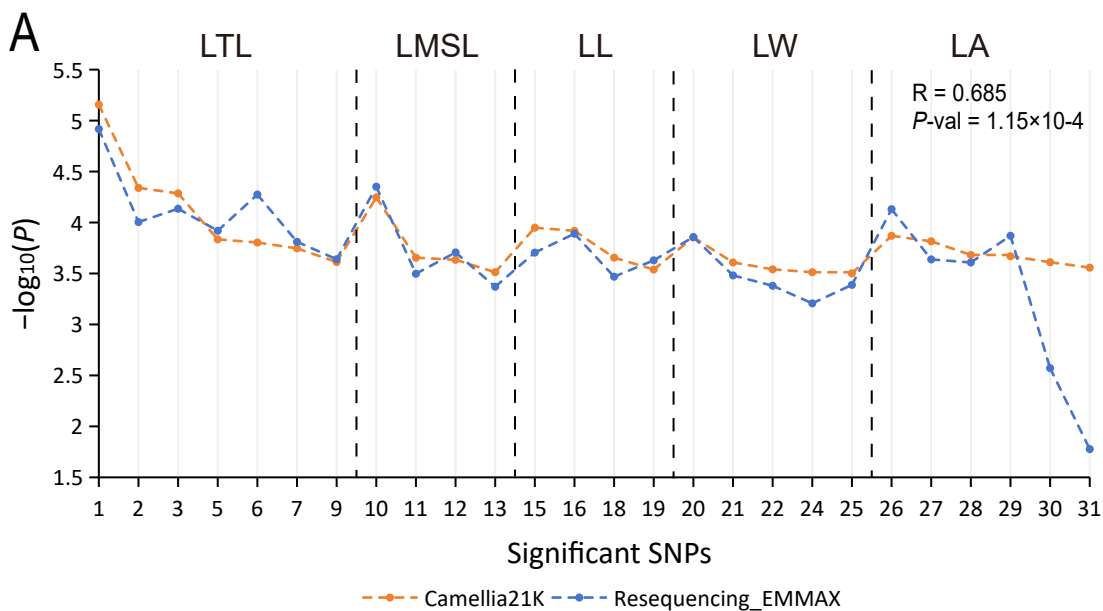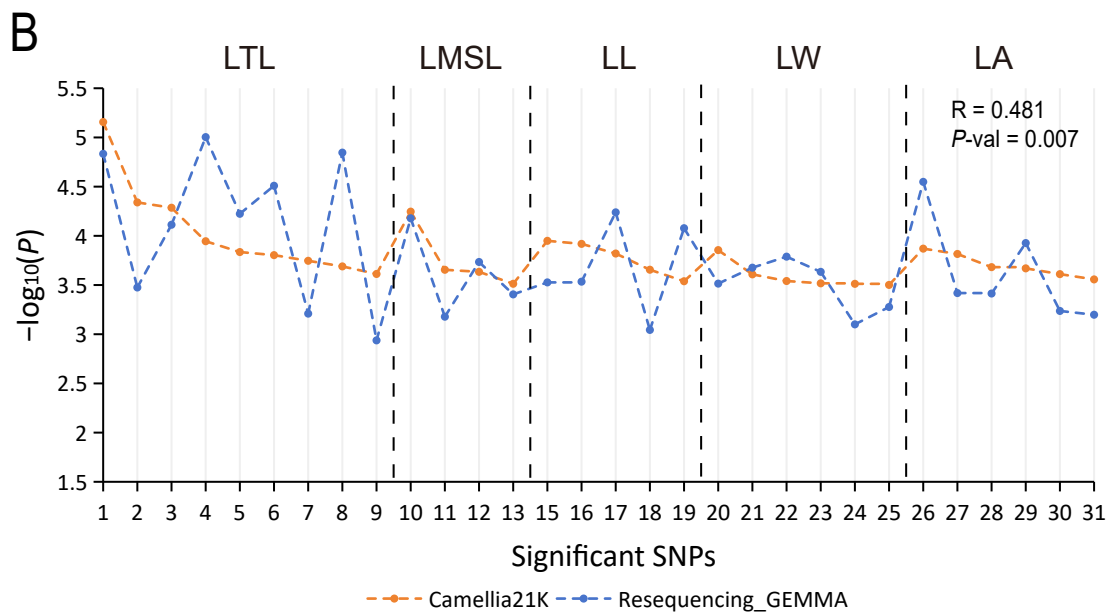

Supplement: Web_Material_uhaf221 [file web_material_uhaf221.zip › fig S3.pdf]
